# What is the mathematical description of the treated mood pattern in bipolar disorder?

**DOI:** 10.3389/fncom.2013.00106

**Published:** 2013-08-12

**Authors:** Fatemeh Hadaeghi, Mohammad R. Hashemi Golpayegani, Shahriar Gharibzadeh

**Affiliations:** Biomedical Engineering Faculty, Amirkabir University of TechnologyTehran, Iran

In their innovative article, Daugherty et al. (2009) have modeled the mood swings of a patient with bipolar disorder as a Liénard oscillator with autonomous forcing. They proposed that emotional state of untreated and treated bipolar type-II patient could be mathematically represented by the Equation (1), in which *x(t)*, represents emotional state in time *t*. In this equation, by adjusting the parameter ρ, both treated and untreated person could be modeled.

(1) $$\begin{array}{c} \ddot{x}-0.38\dot{x}+180x=\rho {\dot{x}}^{3}+\mu {\dot{x}}^{5}-\nu {\dot{x}}^{11} \end{array}$$

**Figure 1:**
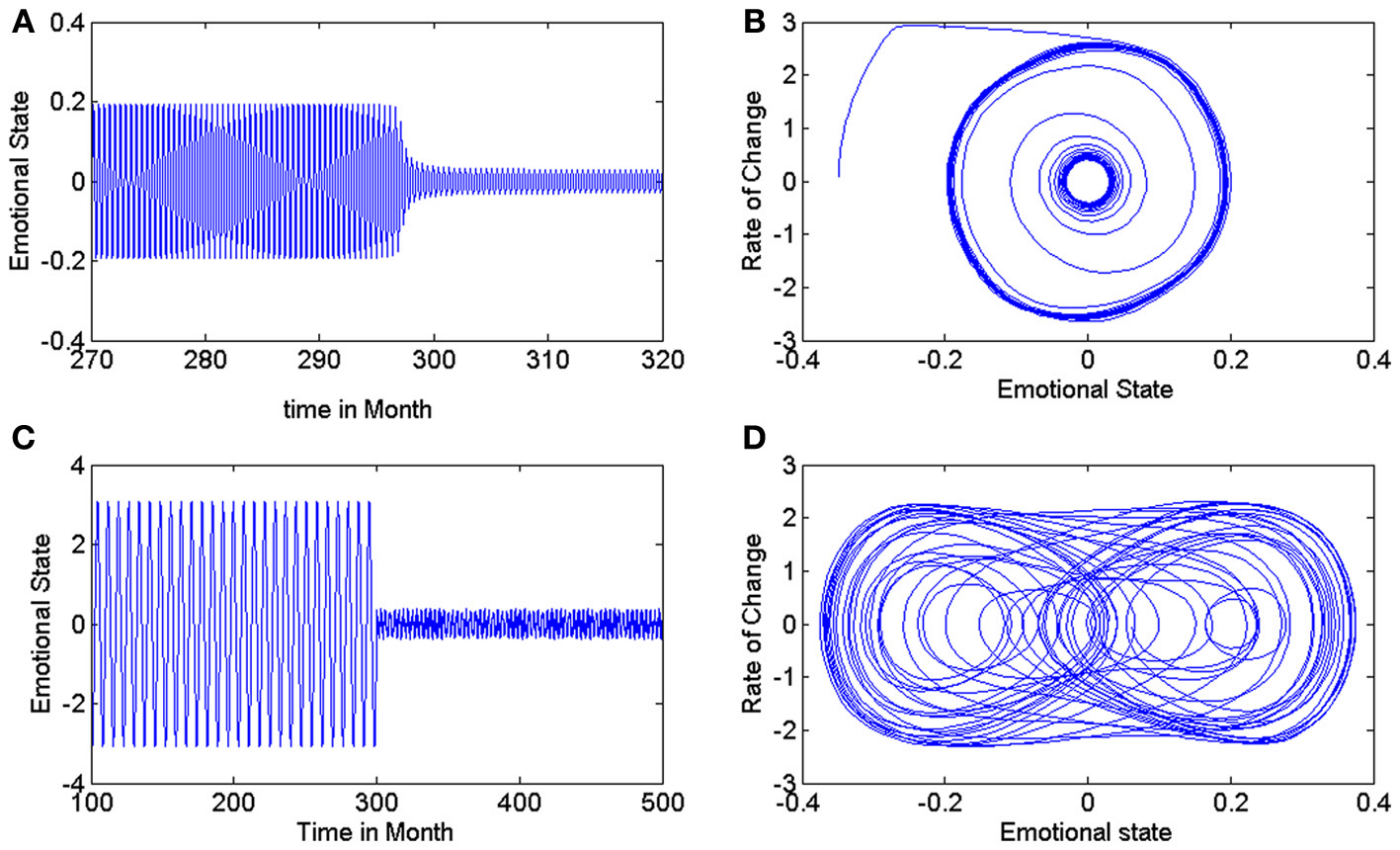
**(A,B)** Time series of mood and phase space of treated patient in model of Equation (1). It has been supposed that smaller stable limit cycle with small amplitude is the desired emotional pattern of the patient after treatment (Daugherty et al., 2009). **(C)** Time series of mood pattern in modified model, before and after treatment. **(D)** Bounded chaotic attractor as a representation of relative variations in emotional state and the rate of its changes in a treated patient using modified model.

The phase space of Equation (1) which is shown in Figure 1B, includes an unstable limit cycle encircled by a large stable limit cycle. The authors have supposed that after treatment, the smaller stable limit cycle with sufficiently small amplitude would correspond to the ultimate emotional pattern to be achieved.

Nevertheless, we believe with basis of previous studies (Gottschalk et al., 1995; Huber et al., 1999) that both in normal persons and treated patients, mood variations and emotional states do not exhibit such a periodic pattern (After 300 months in Figure 1A) and could be better described by a low amplitude chaotic time series. Some of our evidences for this supposition are: (1) the spatial complexity of brain components. In the brain, there are a large number of interacting neurons connected by synapses and interacting networks connected functionally or structurally. As already demonstrated in studies in complex systems, the existence of multiple and interdependent connections acting in complex positive and negative feedback loops is very likely to lead to apparently random and unpredictable states (Korn and Faure, 2003). This unpredictability is a fundamental feature of chaotic patterns. (2) The temporal complexity of brain behavior. Besides the complex structural pattern in the brain, recordings from nerve cells as well as electroencephalograms have showed the chaotic temporal function of the brain in its interaction with the environment (Korn and Faure, 2003; Rabinovich et al., 2012).

In the case of mood as a state of the mind, therefore, it can be expected that mood variation in normal individuals would be more complex rather than being ordered. In addition, the environment is in constant modification and therefore, expecting that it would generate standard and fixed emotional states or moods in such a periodic manner seems to be quite unrealistic. Indeed, in the case of bipolar disorder, it has already been demonstrated that we are dealing with an intermittent behavior (Gottschalk et al., 1995) which can be simplified to a stable periodic pattern, in contrast with the highly chaotic patterns in normal individuals. Therefore, we believe that in treated patients, it would not be adequate to reach a state with periodic oscillation with low amplitude. In fact, in abnormal states, as changes in the complexity of brain dynamics occur, therapeutic strategies would attempt to compensate these changes (Bahrami et al., 2005; Mendez et al., 2012).

Based on the above-mentioned view, we propose to modify the aforementioned model by inserting a time dependent term which reflects the momentary interactions of brain with time varying environment as well as interpersonal relationship. The proposed equation for untreated person could be considered as follows in which ρ = −0.03302, μ = 0.078, ν = 0.00093, and η = 0.1.

(2) $$\ddot{x}-0.038\dot{x}+0.180x=\rho {\dot{x}}^{3}+\mu {\dot{x}}^{5}-\nu {\dot{x}}^{11}-\eta x^{3}$$

The effect of treatments could be inserted through a sinusoidal function which results to Equation (3).

(3) $$\begin{array}{c} \ddot{x}-0.038\dot{x}+0.180x=\rho {\dot{x}}^{3}+\mu {\dot{x}}^{5}-\nu {\dot{x}}^{11}-\eta x^{3} \end{array}+q\cos(\omega t)$$

Changing the parameters of this equation, especially, ω, q, and, η, would yield diverse patterns such as periodic, quasi-periodic, chaotic, and intermittent behaviors. Considering η = 1, ω = 2, and q = 1.2 the Equation (3) has a chaotic solution. In order to provide a deeper insight in to such dynamics, we represent this time series and the chaotic attractor in phase plane in Figures 1C,D. In such example, we present a mathematical representation of an untreated 20-year-old patient Equation (2) as well as the effects of treatment, which is represented by Equation (3). In phase space portrait (Figure 1D), a small amplitude stable chaotic attractor which is encircled by the large unstable periodic orbit (not shown in the figure) represents the desired attractor of emotional state for treated person.

It is obvious that our modified model can represent both rhythmic pattern of mood variation in patients and the complex pattern of mood states in treated subjects. Additionally, our equation seems to be more consistent with observed evidences from empirical studies because its adjustable parameters could reflect the effect of therapeutic strategies (Huber et al., 1999); however, theoretically, the occurrence of a tangent bifurcation in the equation by change in one of the parameters would be required in order to transit from a periodic pattern to a chaotic behavior. The exact meaning of such event in clinical terms still remains to be elucidated in future studies.

Finally, it is important to emphasize that, ultimately, the validity of all these theoretical models and predictions will rely on empirical studies employing qualitative analysis of self-rated mood records (life charts) based on psychological tests or using complexity measures extracted from functional test time series such EEG, fMRI, or PET scan.
